# Revolutionising CKD care: semaglutide’s kidney-protective leap in type 2 diabetes and chronic kidney disease

**DOI:** 10.1097/MS9.0000000000004675

**Published:** 2026-01-14

**Authors:** Syeda Tayyaba, Umair Ali, Iman Osman Abufatima

**Affiliations:** a Department of Medicine, Azad Jammu and Kashmir Medical College, Muzaffrabad, Pakistan; b Department of Pharmacy, University of Swabi, Swabi, Pakistan; c Faculty of Medicine, University of Medical Sciences and Technology, Khartoum, Sudan

*Dear Editor,*

We read with great interest the recent approval of semaglutide by the U.S. Food and Drug Administration for reducing the risk of kidney disease progression and cardiovascular death in adults with type 2 diabetes mellitus (T2DM) and chronic kidney disease (CKD)^[1]^. This milestone expands the therapeutic horizon of GLP-1 receptor agonists beyond glycemic control and weight management, representing a paradigm shift in nephrology and endocrinology. The pivotal FLOW trial demonstrated a 24% relative risk reduction in major kidney outcomes and a 5% reduction in cardiovascular mortality, underscoring the dual renal-cardiac benefit of semaglutide in this high-risk population^[2]^. Mechanistically, GLP-1 receptor agonism has been shown to attenuate hyperfiltration, reduce glomerular injury, and modulate inflammation and oxidative stress, all key contributors to CKD progression in T2DM^[3]^. Prior nephrology therapies predominantly targeted downstream pathways (e.g., RAAS inhibition, SGLT2 blockade) rather than upstream metabolic regulators; hence, semaglutide introduces a novel metabolic-renal axis approach. However, several critical questions remain. The FLOW trial’s median follow-up of approximately 3 years may not fully capture the long-term durability of renal protection or its impact on progression to end-stage kidney disease; real-world evidence and cost-effectiveness analyses will be essential for global adoption^[4]^.

Furthermore, the incremental benefit of semaglutide when used alongside SGLT2 inhibitors and mineralocorticoid receptor antagonists is yet to be established. Notably, an updated meta-analysis by Wanner *et al* revealed that GLP-1 receptor agonists consistently reduce albuminuria and major adverse renal events across diverse CKD cohorts, supporting additive potential with established nephroprotective drugs^[5]^. From a safety standpoint, Johnsson *et al* demonstrated reassuring results regarding tolerability in patients with advanced CKD, showing no unexpected rise in pancreatitis or gastrointestinal adverse events, even with reduced renal clearance^[6]^. Nevertheless, continued pharmacovigilance is warranted to monitor rare adverse events in real-world populations and polypharmacy contexts. Finally, as Packer (2025) eloquently argues, semaglutide’s approval signals a broader paradigm shift; diabetic nephropathy management is moving from renal replacement strategies toward endocrine modulation and metabolic restoration^[7]^. This underscores the urgent need to integrate GLP-1 receptor agonists into future nephrology and endocrinology guidelines, complemented by clinician education and equitable drug access. In conclusion, semaglutide’s kidney-protective indication represents a seminal advance in CKD management. Translating this breakthrough into routine practice through multidisciplinary collaboration, cost accessibility, and long-term surveillance will define the next era of metabolic-renal therapeutics.

This manuscript complies with TITAN Guidelines, 2025, declaring no use of AI^[8]^.

## Data Availability

All data supporting the findings of this study are available within the article. The study is based on previously published literature; no new data were generated or analyzed.
